# Acetazolamide for the prophylaxis of migraine in CADASIL: a preliminary experience

**DOI:** 10.1007/s10194-012-0426-9

**Published:** 2012-02-25

**Authors:** Ida Donnini, Serena Nannucci, Raffaella Valenti, Francesca Pescini, Silvia Bianchi, Domenico Inzitari, Leonardo Pantoni

**Affiliations:** 1 Department of Neurological and Psychiatric Sciences, University of Florence, Largo Brambilla 3, 50134 Florence, Italy; 2 Department of Neurological and Behavioural Sciences, University of Siena, Siena, Italy

**Keywords:** CADASIL, Migraine, Acetazolamide, Prophylaxis, Microangiopathy

## Abstract

Cerebral autosomal dominant arteriopathy with subcortical infarcts and leukoencephalopathy (CADASIL) is an inherited microangiopathy caused by *NOTCH3* mutations. It is characterized by migraine, with or without aura, ischemic events, psychiatric and cognitive disturbances. There is no approved treatment for migraine prophylaxis in CADASIL, but acetazolamide has been anecdotally reported to be effective. We retrospectively reviewed our database of patients with a genetic diagnosis of CADASIL to identify how many of them were treated with acetazolamide for the prophylaxis of migraine. The efficacy and the tolerability of this treatment were checked looking at the clinic reports. Acetazolamide was prescribed in seven patients; the mean duration of treatment was 6 months, and the daily dose ranged from 125 to 500 mg. Three patients had a total and sustained remission, while in two patients a reduction in attacks and an improvement of the headache intensity were recorded. In one of these, acetazolamide was deliberately taken only during the migraine attack and the beneficial effect started 1 h after administration. In two patients, the drug did not produce any beneficial effect. Mild side effects were recorded in two patients. Our preliminary experience expands previous reports and confirms the possible efficacy of acetazolamide in CADASIL migraine. Based on these data, a randomized controlled trial seems worthy to be carried out to test the efficacy and safety of this drug.

## Introduction

Cerebral autosomal dominant arteriopathy with subcortical infarcts and leukoencephalopathy (CADASIL) [MIM 125310] is an autosomal dominant microangiopathy caused by mutations of the *NOTCH3* gene that is located on chromosome 19 and encodes a transmembrane receptor. The clinical spectrum of the disease includes migraine with and without aura, early onset cerebrovascular events, mood disturbances, apathy, cognitive impairment and, more rarely, epilepsy [1, 2]. The typical neuroimaging findings on magnetic resonance imaging (MRI) consist of multifocal and bilateral FLAIR/T2-weighted hyperintensities in the white matter, often involving the anterior temporal poles and the external capsules. Focal hypointensities on T1 (evidence of lacunar infarcts) and lesions suggestive of microhemorrhages in gradient echo images are also seen [3, 4].

A large number of CADASIL patients suffer from migraine. Migraine is frequently the presenting disturbance of the disease, developing in many patients in the second or third decades of life. In CADASIL, migraine with aura is particularly common and a large proportion of patients experience atypical patterns of aura such as aura without headache, basilar and hemiplegic migraine, and attacks of prolonged aura [5]. Recently, cases of persistent aura, status migrainous [6] and acute confusional migraine [7] have also been described in CADASIL patients. Despite migraine is not the most disabling disturbance of CADASIL, it may cause persistent discomfort to patients, especially in those with frequent attacks, and may lead to hospitalization, especially because some auras are difficult to differentiate from cerebrovascular events in these patients. In CADASIL, the coexistent of migraine, cerebrovascular events, and microangiopathy is also a point of interest as far as pathogenesis is concerned.

There have been anecdotic reports on the efficacy of acetazolamide as a treatment of migraine in CADASIL [8, 9], but no further study has explored this aspect. We have thus retrospectively reviewed our database of CADASIL patients to assess how many of them had been treated with acetazolamide for migraine and the efficacy and tolerability of this therapy.

## Methods

For the purpose of our survey, we retrospectively reviewed our database of patients with a genetic diagnosis of CADASIL. The efficacy and the tolerability of acetazolamide treatment were checked looking at the clinic reports. To assess the success of acetazolamide treatment, we evaluated the number and the quality of migraine attacks after starting this prophylactic treatment. We classified as with “significant improvement” all patients in whom no further attack was recorded at subsequent visits and with “improvement” those in whom there was a reduction in intensity and/or frequency of attack, but some disturbances was still recorded. If the file data were incomplete we directly contacted the patients by telephone.

## Results

Our data base includes 51 CADASIL patients. Of these, 32 (63%) had migraine that was classified with aura in 11 of them. Seven patients resulted to have been treated at some points with acetazolamide for migraine prophylaxis (Table 1). Before the introduction of acetazolamide, the mean frequency of migraine attacks was twice a week (ranging from 1 attack/month to a daily disturbance). The duration of migraine attacks differed from one patient to another, and ranged from 30 min up to 1 week. In five of seven patients, migraine started in young age. In all patients, the disturbance worsened over the last years, intensifying its quality and quantity. Two patients started experiencing somatosensory auras after having suffered for many years from migraine without aura. All patients had used some migraine symptomatic treatment, such as paracetamol, non-steroidal anti-inflammatory drugs, or triptans recording different degrees of response. One patient had been on migraine prophylaxis treatment before starting acetazolamide with paroxetine reporting only minimal benefit.

**Table 1:** Patients characteristics, drug treatment, and prophylaxis response

Patient	Sex	Age	Migraine type	*NOTCH3* mutation	Daily dose of acetazolamide (mg)	Duration of treatment	Response	Side effects
1	F	56	Without aura	Exon 2 c.194G > A (p.Cys65Tyr)	250	5 months	None	None
2	F	27	Without aura	Exon 6 c.1012T > C (p.Cys338Arg)	125	6 months	Significant improvement	None
3	F	41	Without aura	Exon 11 c.1819C > T (p.Arg607Cys)	250	6 months	Significant improvement	None
4	F	48	With somatosensory aura and aura without headache (language disturbance)	Exon 19 c.3062A > G (p.Tyr1021Cys)	125	6 years (taken only on migraine attack)	Improvement in 1 h	Subjective gastrointestinal disturbance
5	F	68	Without aura	Exon 11 c.1819 > T (p.Arg607Cys)	500	6 months	None	None
6	F	34	With visual and somatosensory aura	Exon 11 c.1819 > T (p.Arg607Cys)	250	3 months	Significant improvement	None
7	M	56	With somatosensory, visual and language disturbances aura	Exon 19 c.3016C > T (p.Arg1006Cys)	500	1 year	Improvement	Mild increase of creatinine levels

The duration of prophylactic treatment with acetazolamide ranged from 3 to 12 months. Drug treatment was empirically started at low dose (125 mg/day) and gradually increased according to response or side effects. The daily dose ranged from 125 to 500 mg, usually given bid. However, one patient deliberately took the drug only on the occasion of migraine episodes. In five out of seven patients, the treatment was effective with an obvious reduction in the frequency of migraine attacks. In three patients, the benefit could be appreciated within some days of starting of the treatment and produced a total and prolonged remission. In two patients, a reduction in the frequency and severity of attacks was recorded. In the patient taking acetazolamide only during migraine attack, the beneficial effect appeared in about 1 h; her attacks were usually more prolonged. In two patients, the duration and frequency of attacks remained unaltered. When considering side effects, subjective gastrointestinal disturbances were reported by the patient, who took the drug only on the occasion of migraine attacks. This patient also had a diagnosis of celiac disease and severe anxious disturbances. The reported side effects indeed persisted after a first interruption of acetazolamide, and the drug was then started again because of worsening of migraine. In another patient, a mild increase in creatinine levels was recorded and this brought to decrease the daily dose of the drug from 500 to 250 mg.

## Discussion

We have reported our experience with acetazolamide in the treatment of CADASIL migraine. Our preliminary data corroborate two previous single-case reports on the possible efficacy of the drug in this condition [8, 9]. Even if our data derive only from a retrospective database analysis and therefore no definitive conclusion can be derived, it has to be noted that the efficacy of the drug was quite striking with a net benefit reported by five out of seven patients early after drug starting. Surprisingly, the rate of side effects reported by our CADASIL patients appears somehow lower that that expected at least in common migraine [10].

Acetazolamide is a drug used for a variety of conditions such as congestive heart failure, some forms of epilepsy, glaucoma, idiopathic intracranial hypertension, but also rare diseases, such as acute mountain sickness [11].

When considering central nervous system effects, acetazolamide penetrates the blood–brain barrier slowly, causes carbonic acidosis through carbonic anhydrase inhibition [12], and induces in normal subjects a considerable increase in cerebral blood flow by vasodilation. Because of this effect, acetazolamide infusion is also used to evaluate the degree of alterations in cerebrovascular reactivity [13, 14]. This effect may depend on a metabolic change induced by the enzyme blockage through a decrease in perivascular pH and increasing the concentration of nitric oxide or also by a direct effect on the wall of small brain arterioles and arteries [15], the real underlying mechanism still being unclear [16, 17].

The acetazolamide-dependent cerebral vasoreactivity has been also assessed in CADASIL. In this disease, the drug is able to produce an increase in cerebral perfusion as seen by transcranial Doppler sonography [18], perfusion MRI [19] or Tc-99m ECD brain perfusion SPECT [20].

One study has investigated acetazolamide efficacy in sporadic migraine with and without aura, demonstrating a reduction in the frequency of attacks in both migraine types [21]. This effect is explained by two possible mechanisms. The first one sees migraine deriving from cerebral oligemia [22, 23], and acetazolamide acting through vasodilatation. The second attributes migraine to a possible disorder of neuronal ion channels [21]. This last mechanism is the one thought to be responsible for the acetazolamide mechanism of action in familial ion channels disorders, such as familial hemiplegic migraine, hypokalemic periodic paralysis and episodic ataxia type 2 [24–26]. One can hypothesize that one of the above-mentioned mechanisms or even both are present also in CADASIL migraine.

The presence of migraine in CADASIL leads to some considerations [27]. Today, CADASIL migraine is classified according to the International Classification of Headache Disorders (ICHD-2) as Headache attributed to cranial or cervical vascular disorder. This implies that this disturbance has a vascular basis. However, it is known that, in this disease, migraine symptoms precede the onset of ischemic events by several years, and that the typical CADASIL strokes are mostly of the subcortical type and associated with lacunar syndromes, whereas migraine-associated disturbances, such as dysphasia and visual aura are linked with cortical phenomena [5]. One possible explanation for this seemingly discrepancy in CADASIL could be to hypothesize the presence of an ultrastructural smooth muscle cells modification in the meningeal and cortical vessels [7]. A second hypothesis sees the presence of a possible neuronal cell dysfunction, such as the above-reported changes in calcium channels or in glial sodium channels (seen also in other hereditary diseases like familial hemiplegic migraine and mitochondrial disorders) that have been already reported to be related to a higher level of brain hyperexcitability [28], suggesting a potential role in CADASIL migraine. The complexity of the migraine mechanism in CADASIL is also supported by the recently reported efficacy of intravenous sodium valproate in one patient with a combination of seizures and migraine [29].

In conclusion, our data seem to hint at a significant effect of acetazolamide in CADASIL migraine prophylaxis. Our small series of CADASIL patients expands the previous literature on the topic, but clearly it is numerically very limited and retrospective in nature, and therefore no final conclusion about the use of acetazolamide in CADASIL migraine can be drawn at present. An appropriately sized randomized, double-blind, trial is therefore needed. When considering the absence of data and the severity of the disturbance in CADASIL patients, such a trial appears worthy to be started. In our opinion, two other reasons make a trial of this type potentially appealing. First, the systematic and controlled use of acetazolamide in CADASIL could indirectly provide some information about the pathogenesis of the disease, particularly on the role of microvessels. Second, a trial with acetazolamide could also provide some information about the possible effect of the drug on other disease disturbances. Of note, one preliminary study has already shown that a 24-week administration of acetazolamide is able to improve cerebral hemodynamics in CADASIL [18].

## References

[1] Chabriat H, Joutel A, Dichgans M, Tournier-Lasserve E, Bousser MG (2009). CADASIL. Lancet Neurol.

[2] Reyes S, Viswanathan A, Godin O, Dufouil C, Benisty S, Hernandez K, Kurtz A, Jouvent E, O’Sullivan M, Czernecki V, Bousser MG, Dichgans M, Chabriat H (2009). Apathy: a major symptom in CADASIL. Neurology.

[3] Chabriat H, Mrissa R, Levy C, Vahedi K, Taillia H, Iba-Zizen MT, Joutel A, Tournier-Lasserve E, Bousser MG (1999). Brain stem MRI signal abnormalities in CADASIL. Stroke.

[4] André C (2010). CADASIL: pathogenesis, clinical and radiological findings and treatment. Arq Neuropsiquiatr.

[5] Vahedi K, Chabriat H, Levy C, Joutel A, Tournier-Lasserve E, Bousser MG (2004). Migraine with aura and brain magnetic resonance imaging abnormalities in patients with CADASIL. Arch Neurol.

[6] Sacco S, Rasura M, Cao M, Bozzao A, Carolei A (2009). CADASIL presenting as status migrainosus and persisting aura without infarction. J Headache Pain.

[7] Sathe S, DePeralta E, Pastores G, Kolodny EH (2009). Acute confusional migraine may be a presenting feature of CADASIL. Headache..

[8] Weller M, Dichgans J, Klockgether T (1998). Acetazolamide-responsive migraine in CADASIL. Neurology.

[9] Forteza AM, Brozman B, Rabinstein AA, Romano JG, Bradley WG (2001). Acetazolamide for the treatment of migraine with aura in CADASIL. Neurology..

[10] Vahedi K, Taupin P, Djomby R, El-Amrani M, Lutz G, Filipetti V, Landais P, Massiou H, Bousser MG (2002). Efficacy and tolerability of acetazolamide in migraine prophylaxis: a randomised placebo-controlled trial. J Neurol.

[11] Leaf DE, Goldfarb DS (2007). Mechanisms of action of acetazolamide in the prophylaxis and treatment of acute mountain sickness. J Appl Physiol.

[12] Settakis G, Molnár C, Kerényi L, Kollár J, Legemate D, Csiba L, Fülesdi B (2003). Acetazolamide as a vasodilatory stimulus in cerebrovascular diseases and in conditions affecting the cerebral vasculature. Eur J Neurol.

[13] Yonas H, Darby JM, Marks EC, Durham SR, Maxwell C (1991). CBF measured by Xe-CT: approach to analysis and normal values. J Cereb Blood Flow Metab.

[14] Eskey CJ, Sanelli PC (2005). Perfusion imaging of cerebrovascular reserve. Neuroimaging Clin N Am.

[15] Ridderstråle Y, Hanson M (1985). Histochemical study of the distribution of carbonic anhydrase in the cat brain. Acta Physiol Scand.

[16] Gambhir S, Inao S, Tadokoro M, Nishino M, Ito K, Ishigaki T, Kuchiwaki H, Yoshida J (1997). Comparison of vasodilatory effect of carbon dioxide inhalation and intravenous acetazolamide on brain vasculature using positron emission tomography. Neurol Res.

[17] White RP, Deane C, Vallance P, Markus HS (1998). Nitric oxide synthase inhibition in human reduces cerebral blood flow but not the hyperemic response to hypercapnia. Stroke.

[18] Huang L, Yang Q, Zhang L, Chen X, Huang Q, Wang H (2010). Acetazolamide improves cerebral hemodynamics in CADASIL. J Neurol Sci.

[19] Chabriat H, Pappata S, Ostergaard L, Clark CA, Pachot-Clouard M, Vahedi K, Jobert A, Le Bihan D, Bousser MG (2000). Cerebral hemodynamics in CADASIL before and after acetazolamide challenge assessed with MRI bolus tracking. Stroke.

[20] Park SA, Yang CY, Choi SS, Kim WH (2011). Assessment of cerebral hemodynamics to acetazolamide using brain perfusion SPECT in cerebral autosomal dominant arteriopathy with subcortical infarcts and leukoencephalopathy. Clin Nucl Med.

[21] De Simone R, Marano E, Di Stasio E, Bonuso S, Fiorillo C, Bonavita V (2005). Acetazolamide efficacy and tolerability in migraine with aura: a pilot study. Headache.

[22] Sacco S, Degan D, Carolei A (2010). Diagnostic criteria for CADASIL in the International Classification of Headache Disorders (ICHD-II): are they appropriate?. J Headache Pain.

[23] Skyhøj Olsen T (1990). Migraine with and without aura: the same disease due to cerebral vasospasm of different intensity. A hypothesis based on CBF studies during migraine. Headache.

[24] Robbins MS, Lipton RB, Laureta EC, Grosberg BM (2009). CACNA1A nonsense mutation is associated with basilar-type migraine and episodic ataxia type 2. Headache.

[25] Tricarico D, Barbieri MG, Conte Camerino D (2000). Acetazolamide opens the muscular KCa^2+^ channel: a novel mechanism of action that may explain the therapeutic effect of the drug in Hypokalemic Periodic Paralysis. Ann Neurol.

[26] Omata T, Takanashi J, Wada T, Arai H, Tanabe Y (2011). Genetic diagnosis and acetazolamide treatment of familial hemiplegic migraine. Brain Dev.

[27] Liem MK, Oberstein SA, van der Grond J, Ferrari MD, Haan J (2010). CADASIL and migraine: a narrative review. Cephalalgia.

[28] Speciali JG, Bigal ME (2006). Subcortical lesions in migraine: are they related to mitochondrial dysfunction?. Headache.

[29] Martikainen MH, Roine S (2012). Rapid improvement of a complex migrainous episode with sodium valproate in a patient with CADASIL. J Headache Pain.

